# Editorial: The role of bone-muscle crosstalk in secondary osteoporosis

**DOI:** 10.3389/fendo.2024.1454743

**Published:** 2024-08-21

**Authors:** Lorenzo Lippi, Alessandro de Sire, Marco Invernizzi

**Affiliations:** ^1^ Department of Scientific Research, Off-Campus Semmelweis University of Budapest, Budapest, Hungary; ^2^ Physical and Rehabilitative Medicine, Department of Medical and Surgical Sciences, University of Catanzaro “Magna Graecia”, Catanzaro, Italy; ^3^ Research Center on Musculoskeletal Health, MusculoSkeletalHealth@UMG, University of Catanzaro “Magna Graecia”, Catanzaro, Italy; ^4^ Physical and Rehabilitative Medicine, Department of Health Sciences, University of Eastern Piedmont “A. Avogadro”, Novara, Italy; ^5^ Dipartimento Attività Integrate Ricerca e Innovazione (DAIRI), Translational Medicine, Azienda Ospedaliera SS. Antonio e Biagio e Cesare Arrigo, Alessandria, Italy

**Keywords:** osteoporosis, sarcopenia, telemedicine, rehabilitation, precision medicine

Secondary osteoporosis is a multifactorial condition, frequently linked to chronic inflammation, hormonal dysregulation, pharmacological treatments, and lifestyle behaviors (1). Managing bone health in these patients is a critical issue, requiring a comprehensive understanding of different contributing factors (**Figure 1** shows potential contributing factors for secondary osteoporosis).

**Figure 1:**
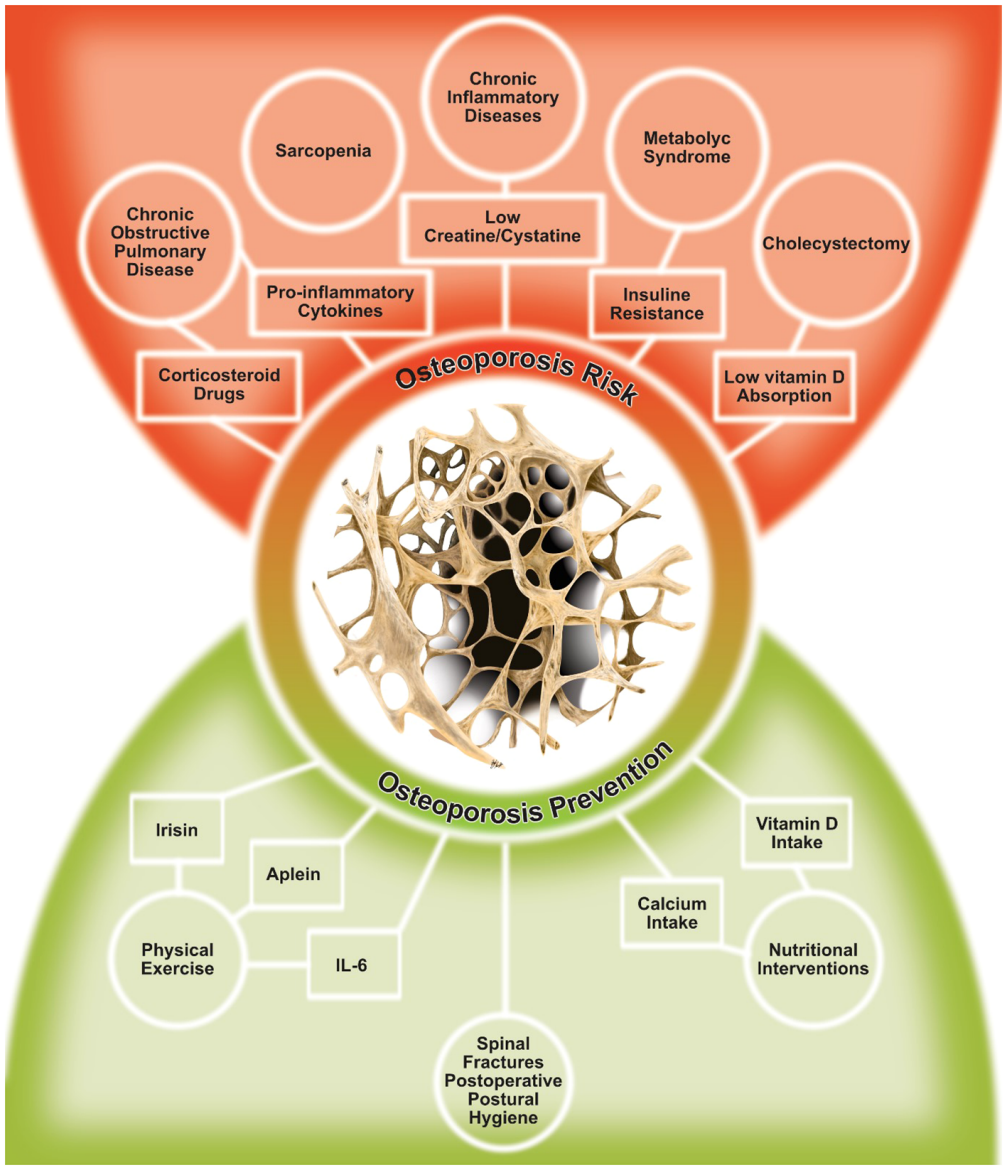
Risk factors for secondary osteoporosis and potential targets for osteoporosis prevention.

Osteoporosis and sarcopenia are interconnected conditions that might synergically contribute to increase the risk of falls, fractures, and subsequent physical impairment. Recognizing the bidirectional relationship between bone and muscle health is essential for developing effective therapeutic strategies in patients with secondary osteoporosis.

In this Research Topic, we present the critical role of bone-muscle crosstalk, providing an overview of recent research highlighting the interactions between bone and muscle and emphasizing the need for integrated care approaches.

Interestingly, clinical biomarkers have been fully integrated into the clinical management of patients with secondary osteoporosis for both monitoring disease progression and evaluating the efficacy of treatments, but few data are currently available on suitable biomarkers evaluating secondary osteoporosis risk. The recent study by He et al. demonstrated that a reduced creatinine-cystatin C ratio (CCR) is associated with an increased risk of osteoporosis and fractures, highlighting a close link between muscle and bone health. Indeed, CCR is a pivotal biomarker for assessing muscle health and is important in sarcopenia assessment due to its relations with muscle mass and function. This study provided additional data about the intricate interplay between muscle and bone, suggesting that bone-muscle crosstalk represents a potential target for advancing personalized care and improving patient outcomes in clinical practice.

In this context, sarcopenia is highly prevalent among patients with secondary osteoporosis, with detrimental implications for both the risk of falls and the risk of fracture. On the other hand, strong evidence is available on the detrimental consequences of fragility fractures in muscle quality and the musculoskeletal system. Accordingly, the study by Huang et al. (2) provides valuable insights into the stress distribution within osteoporotic spines which might be crucial for developing post-operative management strategies aimed at preventing secondary fractures by avoiding prolonged spinal curvature postures. These data might be crucial for the rehabilitation management of fragility fractures, providing further insight for a precise assessment of patients with osteoporosis.

Considering the functional, embryological, and biochemical connections between bone and muscle tissues, recent literature is focusing on the bone-muscle crosstalk as a potential target for precise approaches to fragility fractures. Interestingly, growing literature is currently emphasizing the role of physical exercise in stimulating bone quality and bone mineral density in patients with disabling conditions. More in detail, performing physical exercise, osteoblasts release undercarboxylated osteocalcin, which improves muscle function. The recent study by Zhao et al. has shown that exercise enhances bone strength, releasing IGF-1 into the bloodstream, and also facilitates the release of myostatin, IL-6, irisin, and apelin from muscles into the bloodstream, thereby influencing bone remodeling to maintain a balance between bone absorption and formation.

Nutritional interventions also play a pivotal role in managing osteosarcopenia. The role of insulin resistance, osteoporosis, and sarcopenia is controversial and complex. Insulin resistance, commonly associated with metabolic syndrome, has been implicated in both reduced bone mineral density (BMD) and muscle wasting. In the study by Fu et al. the authors assessed data from 5,292 participants in the National Health and Nutrition Examination Survey, researchers found that insulin resistance (HOMA-IR) and β-cell function (HOMA-β) are associated with bone mineral density (BMD) and osteoporosis. The study revealed a positive association between high insulin resistance and osteoporosis in participants with high β-cell function, and a negative association between high β-cell function and osteoporosis in participants with low insulin resistance. These findings suggest that the relationship between β-cell function and osteoporosis varies depending on the level of insulin resistance.

In conclusion, the interconnections between inflammatory conditions, osteoporosis, and sarcopenia form a complex web of health challenges that significantly impact the quality of life and prognosis of affected individuals. Recognizing and addressing osteosarcopenia as a distinct entity in inflammatory disorders is crucial for developing effective, comprehensive management strategies. By integrating rehabilitation, nutritional support, pharmacological treatments, and early diagnostic measures, healthcare providers can break the cycle of decline and improve outcomes for patients grappling with these intertwined conditions. Therefore, it is mandatory a holistic approach not only targeting systemic inflammation but also addressing the broader musculoskeletal health to ensure better overall health and quality of life for patients with inflammatory disorders.

## References

[1] de Sire A Lippi L Aprile V Calafiore D Folli A D’Abrosca F. Pharmacological, nutritional, and rehabilitative interventions to improve the complex management of osteoporosis in patients with chronic obstructive pulmonary disease: a narrative review. J Personalized Med. (2022) 12:1626. doi: 10.3390/jpm12101626 PMC9604650 36294765

[2] Huang S Zhou C Zhang X Tang Z Liu L Meng X. Biomechanical analysis of sandwich vertebrae in osteoporotic patients: finite element analysis. Front Endocrinol (Lausanne). (2023) 14:1259095. doi: 10.3389/fendo.2023.1259095 37900139 PMC10600377

